# Identification of Tendency to Alcohol Misuse From the Structural Brain Networks

**DOI:** 10.3389/fnsys.2020.00009

**Published:** 2020-03-03

**Authors:** Sujung Yoon, Jungyoon Kim, Gahae Hong, Tammy D. Kim, Haejin Hong, Eunji Ha, Jiyoung Ma, In Kyoon Lyoo

**Affiliations:** ^1^Ewha Brain Institute, Ewha W. University, Seoul, South Korea; ^2^Department of Brain and Cognitive Sciences, Ewha W. University, Seoul, South Korea; ^3^Graduate School of Pharmaceutical Sciences, Ewha W. University, Seoul, South Korea; ^4^The Brain Institute and Department of Psychiatry, University of Utah, Salt Lake City, UT, United States

**Keywords:** alcohol misuse, impulsivity, reward sensitivity, avoidance, anger, addiction circuitry, structural brain network

## Abstract

The propensity to engage in risky behaviors including excessive alcohol consumption may impose increased medical, emotional, and psychosocial burdens. Personality and behavioral traits of individuals may contribute in part to the involvement in risky behaviors, and therefore the classification of one’s traits may help identify those who are at risk for future onset of the addictive disorder and related behavioral issues such as alcohol misuse. Personality and behavioral characteristics including impulsivity, anger, reward sensitivity, and avoidance were assessed in a large sample of healthy young adults (*n* = 475). Participants also underwent diffusion tensor imaging for the analysis of structural brain networks. A data-driven clustering using personality and behavioral traits of the participants identified four subtypes. As compared with individuals clustered into the neutral type, individuals with a high level of impulsivity (A subtype) and those with high levels of reward sensitivity, impulsivity, anger, and avoidance (B subtype) showed significant associations with problem drinking. In contrast, individuals with high levels of impulsivity, anger, and avoidance but not reward sensitivity (C subtype) showed a pattern of social drinking that was similar to those of the neutral subtype. Furthermore, logistic regression analysis with ridge estimators was applied to demonstrate the neurobiological relevance for the identified subtypes according to distinct patterns of structural brain connectivity within the addiction circuitry [neutral vs. A subtype, the area under the receiver operator characteristic curve (AUC) = 0.74, 95% CI = 0.67–0.81; neutral vs. B subtype, AUC = 0.74, 95% CI = 0.66–0.82; neutral vs. C subtype, AUC = 0.77, 95% CI = 0.70–0.84]. The current findings enable the characterization of individuals according to subtypes based on personality and behavioral traits that are also corroborated by neuroimaging data and may provide a platform to better predict individual risks for addictive disorders.

## Introduction

Addictive behaviors in everyday life may be frequently associated with negative consequences on health and psychological well-being (Deleuze et al., 2015). While the debate over the relationship between personality traits and behavioral outcome continues, recent trends focus on the role of particular behavioral and personality characteristics in the propensity to engage in addictive behaviors as well as vulnerability to the future onset of addictive disorders (Berglund et al., 2011; Whelan et al., 2014; Egervari et al., 2018). For instance, growing literature suggest the importance of personality traits in addiction, such as the role of personality in the vulnerability or resilience towards substance use disorders (Belcher et al., 2014) and addictive behaviors (Barkin et al., 2002), as well as the identification of specific traits that have been demonstrated as prevalent in substance users (Terracciano et al., 2008). In particular, traits such as impulsivity (de Wit, 2009; Ersche et al., 2012a,b) and reward sensitivity (Ersche et al., 2013) have been previously reported in association with substance use. Furthermore, structural alterations of specific brain regions may act as neural correlates of addictive behaviors, such as response inhibition (Nigg et al., 2006) and neurobehavioral disinhibition (Tarter et al., 2003) which have been demonstrated as a potential predictor for problem drinking and substance use disorder, respectively. Considering this, an investigational approach that can reliably identify at-risk individuals may be beneficial in the assessment of individual prognosis for addictive disorders, and necessary in building personalized preventive and intervention strategies.

Recent advances in neuroimaging and computational techniques greatly enhanced our understanding of the human brain and behavior and enabled the quantification of their variations across individuals in both healthy and disease conditions (Gabrieli et al., 2015). Studies revealed possibilities for the discovery of brain measures and their correlation with our predictive ability in health-related behaviors (Helfinstein et al., 2014; Gabrieli et al., 2015; Poldrack et al., 2018). Furthermore, recent large-scale longitudinal studies on adolescents demonstrated that individual personality and cognitive differences, environmental factors, and candidate genes along with brain structural and functional characteristics not only identified current alcohol misuse but also predicted future alcohol misuse (Whelan et al., 2014; Heinrich et al., 2016).

Increasing evidence suggests that the neuropathological features of a set of brain regions that make up the “addiction circuitry” play an important role in the development and progression of addictive disorders (Koob and Volkow, 2016). According to previous research, abnormalities in individual brain regions, as well as the interconnections within the addiction circuitry, may be considerably involved in addictive behaviors (Koob and Volkow, 2016). Likewise, a series of recent studies on structural brain networks demonstrated alterations in interconnections of brain regions of the addiction circuitry in relation to alcohol dependence and other forms of substance dependence (Lim et al., 2002; Romero et al., 2010; Ersche et al., 2012a,b; Zhang et al., 2016). As such, following the recent trend of applying a connectome-based approach may be useful in identifying the distinct brain correlates that are reflective of various personality and behavioral traits in relation to risks for alcohol misuse.

The present study aims to identify distinct subtypes according to the observable behavioral characteristics in a large non-clinical sample of young adults and to examine whether these subtypes were related to alcohol drinking patterns. Behavioral and personality characteristics including impulsivity, anger, reward-sensitivity, and avoidance, all of which have been reported as being associated with the vulnerability to addictive behaviors (Castellanos-Ryan et al., 2013; Egervari et al., 2018), were included in this data-driven model. In addition, in an effort to provide reliable neurobiological correlates of this clinical data-based clustering, further validation steps included the classification of each individual into a particular subtype according to one’s neurobiological measures, such as white matter fiber interconnections among the brain regions of the addiction circuitry.

We hypothesized that individuals who are categorized under a distinct subtype based on behavioral traits would show type-specific neurobiological abnormalities as observed by neuroimaging data, including altered white matter fiber interconnections within the addiction circuitry.

## Materials and Methods

### Participants

A total of 475 young adults [387 men and 88 women; mean age ± standard deviation (SD) 27.1 ± 3.7 years old] who were screened for the absence of any major medical or neuropsychiatric diseases using medical history taking and the Structured Clinical Interview for DSM-4 (First et al., 1997), respectively, were recruited from the community. Individuals were excluded if they had any past or current alcohol or substance dependence, were shown positive on the pregnancy test, had a history of traumatic brain injury with loss of consciousness, or had any contraindications to brain magnetic resonance imaging (MRI).

All participants underwent a two-part evaluation: (1) clinical assessments of addiction-related behavioral and personality characteristics including alcohol misuse, impulsivity, anger, reward-sensitivity, and avoidance; and (2) neuroimaging assessments of structural brain networks.

This study protocol was approved by the Institutional Review Board of Ewha W. University. Written informed consent was obtained from all participants prior to enrollment.

**Figure 1:**
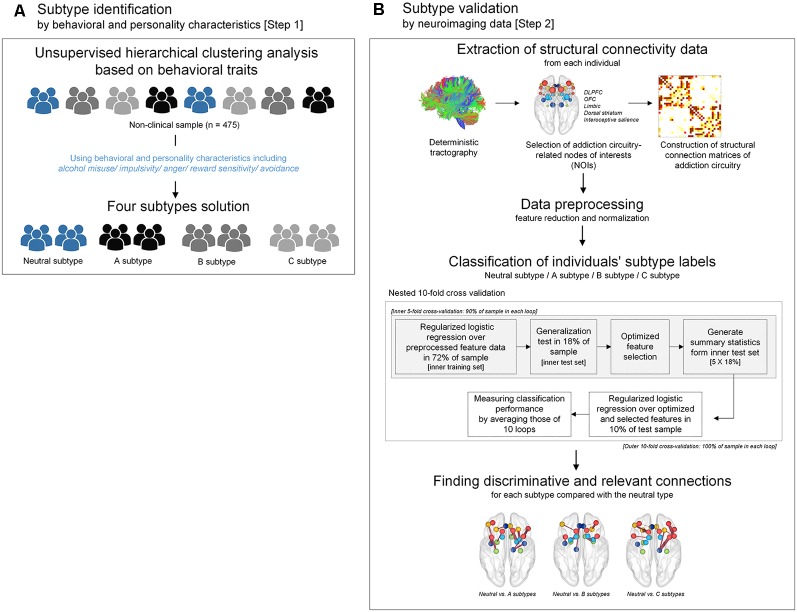
A flow chart demonstrating the conceptual approach of the study design including subtype identification and validation. **(A)** Subtypes were identified by unsupervised hierarchical clustering analysis using scale scores of alcohol misuse, impulsivity, anger, reward sensitivity, and avoidance. **(B)** Subtypes that were clustered according to behavioral and personality characteristics were validated by a supervised approach using structural connectivity data of the addiction circuitry encompassing of DLPFC, OFC, limbic structure, dorsal striatum, and interoceptive salience processing brain regions. Logistic regression analysis with ridge estimators was applied to predict each individual’s subtype under the framework of nested 10-fold cross-validation. DLPFC, dorsolateral prefrontal cortex; OFC, orbitofrontal cortex.

In the current study, unsupervised hierarchical clustering analysis was performed to identify particular subtypes related to addictive behaviors according to similarities in behavioral and personality characteristics (Step 1 subtype identification in Figure 1). The most discriminative connection features for classifying each subtype were then selected using logistic regression analysis with ridge estimators in order to provide the neurobiological relevance for these identified subtypes (Step 2 subtype validation in Figure 1).

### Behavioral and Personality Assessments

Addiction-related behavioral and personality characteristics were assessed using five scales including the Alcohol Use Disorders Identification Test (AUDIT; Saunders et al., 1993), Barratt impulsiveness scale (BIS; Barratt, 1994; Patton et al., 1995), State-Trait Anger Expression Inventory (STAXI; Spielberger, 1988), Behavioral Inhibition System scale (Carver and White, 1994) and Behavioral Approach System scale (Carver and White, 1994).

As supplementary measures, personality characteristics were assessed using the personality diagnostic questionnaire-4+ (PDQ-4+; Hyler, 1994), and composite scores for two personality tendencies which are extroverted and anxious-depressive characteristics were calculated and used for further analyses.

See Supplementary Methods for details of each measure.

### Unsupervised Clustering of Individuals According to Behavioral and Personality Measures

**Figure 2:**
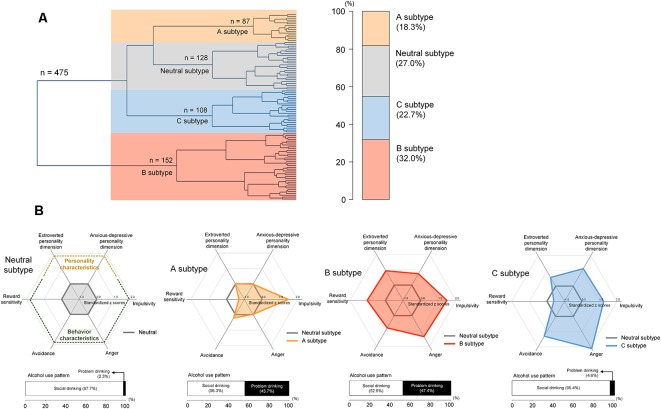
Hierarchical clustering of a sample into distinct subtypes according to behavioral and personality characteristics including alcohol misuse, impulsivity, anger, reward sensitivity, and avoidance **(A)** and scale scores for these characteristics in each subtype **(B)**. The subtypes were defined as the neutral subtype (*n* = 128), A subtype (*n* = 87), B subtype (*n* = 152), and C subtype (*n* = 108). Values in the radar charts were transformed into standardized z scores using the means and standard deviations of the scale scores from the neutral subtype. Stacked horizontal bar graphs in panel **(B)** indicate that, as compared with those under the neutral subtype, individuals with A and B subtypes may display more alcohol-related problems. There are no differences in the frequency of problem drinking between the neutral and C subtypes.

Agglomerative hierarchical clustering analysis was performed on the standardized scores of the five abovementioned scales on addiction-related behavioral and personality characteristics to determine the number of significant clusters and identify clustered subtypes. Using Ward’s minimum variance method while considering the squared Euclidean distance (Ward, 1963), a dendrogram strongly suggested a 4-cluster solution (Figure 2). This 4-cluster solution was considered most optimal for discriminating participants into clusters that are maximally dissimilar from each other. We also ensured that the 4-cluster solution could be theoretically interpreted while having each individual cluster consist of at least 20% of the total participants. Discriminant function analysis with a leave-one-out cross-validation was applied to validate the identified optimal clustering solution, which yielded a high accuracy of 80.2% across clusters. The process of the identification subtypes is conceptually summarized in Figure 1A.

### Construction of Structural Connection Matrix of Addiction Circuitry and Group Comparisons

For the construction of structural brain connectivity matrix, high-resolution T1-weighted and diffusion-weighted images were acquired using a 3.0 Tesla whole-body imaging system. Diffusion tensor was calculated using the Diffusion Toolkit^1^, and white matter pathways within the addiction circuitry were reconstructed using the Fiber Assignment by Continuous Tracking (FACT) algorithm (Mori et al., 1999). The Trackvis^2^ software package was applied to reconstruct all fiber tracts that were interconnecting the nodes of interest (NOIs). See Supplementary Methods for a detailed description of the image data acquisition and preprocessing.

**Figure 3:**
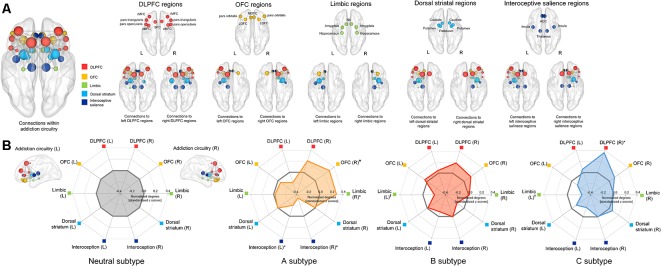
Schematic representation of the group-averaged reconstructed structural brain network among the brain regions (nodes) related to the addiction circuitry, which were then grouped into 5 ROIs including the DLPFC, OFC, limbic, dorsal striatal, and interoceptive salience processing regions **(A)** and radar charts for the normalized network connections (degrees) of each ROI in each subtype **(B)**. For the purpose of presentation, the spheres are colored according to the ROIs to which they belong, and are placed in the center of each brain region (node) as according to the Desikan-Killiany atlas. The node size is proportional to the number of network connections (degree). Values of normalized degrees were transformed into standardized z scores using the means and standard deviations of those from the neutral subtype. Asterisks (*) in the radar charts indicate significant differences in normalized degrees of each subtype as compared with those in the neutral subtype at permutation-adjusted *P* < 0.05. For presentation purposes, a marginal statistical significance of group differences in normalized degrees at permutation-adjusted *P* < 0.09 was also noted using crosses (†). DLPFC, dorsolateral prefrontal cortex; rMFC, rostral medial frontal cortex; SFC, superior frontal cortex; cMFC, caudal medial frontal cortex; OFC, orbitofrontal cortex; LOFC, lateral orbitofrontal cortex; MOFC, medial orbitofrontal cortex; NA, nucleus accumbens; ACC, anterior cingulate cortex; L, left; R, right.

A total of 34 brain regions (17 regions per each hemisphere) were identified as regions that are part of the addiction circuitry and involved in the pathophysiology of addictive disorders (Koob and Volkow, 2016; Volkow et al., 2016). These regions were defined as network NOIs of the addiction circuitry based on individual parcellation maps according to the Desikan-Killiany atlas (Desikan et al., 2006) generated using the FreeSurfer software suite^3^. The NOIs were grouped into five regions of interest (ROIs) according to their anatomical locations and functions as follows: the dorsolateral prefrontal cortical region (DLPFC), orbitofrontal cortical region (OFC), limbic region, dorsal striatal region, and interoceptive salience processing region, all bilaterally (Figure 3A). Individual brain regions of each ROI are presented in Supplementary Table S1. Brain regions clustered into the DLPFC ROI are known to be involved in functions of executive control related to addiction, while those into the OFC and limbic ROIs may play an important role in the processing of withdrawal-negative effect related to addiction (Koob and Volkow, 2016). The reward and incentive salience processing related to addiction may be modulated by brain regions of the dorsal striatal and interoceptive salience processing ROIs (Koob and Volkow, 2016). Each structural connection (edge) between two NOIs was defined as having a minimum of three interconnected streamlines, which were measured using the deterministic fiber tracking method (Yoon et al., 2016). For each individual, a 17 × 17 structural connection matrix was constructed per hemisphere. The strength of the connections between each pair of NOIs was measured using the number of streamlines connected and were entered into the classification models as relevant connection features. A total of 272 bilateral intra-hemispheric connections were then used as connection feature candidates.

The degree of each NOI was defined as the number of connections to other NOIs (Rubinov and Sporns, 2010), and was normalized by the total number of connections within the reconstructed matrix. The sum of the normalized degree of NOIs was calculated for each of the five ROIs and values for each subtype are presented in Supplementary Table S2. The normalized degrees of ROIs were compared between the groups using the general linear model with sex as a relevant covariate. Permutation-adjusted *P* values were calculated (10,000 permutations) in order to correct for multiple comparisons (Westfall and Young, 1993).

### Subtype Validation: Model Construction for Classifying Identified Subtypes Using Neuroimaging Data

#### Feature Processing and Reduction

Bilateral intra-hemispheric connection features of the addiction circuitry were normalized within the range of 0–1 in order to minimize noise-related scaling effects. Among 272 connection features, the top 20% of connection features (*n* = 54) that were identified as being different compared to the neutral subtype remained for each classification model. A total of 54 connection features remained after the initial feature reduction, and are listed in Supplementary Table S3.

#### Feature Selection, Classification Algorithm, and Evaluation of Classification Performance

In addition to the initial feature reduction, suboptimal and redundant connection features were subsequently removed using a feature selection algorithm based on the wrapper approach in order to avoid the overfitting problem (Friedman et al., 2001). As compared with the filter methods for feature selection, nested cross-validation based on the wrapper approach would have the benefit of efficient control of the multicollinearity issue among features (Mwangi et al., 2014), which is a prevalent concern in neuroimaging data due to its high dimensionality.

Nested cross-validation with the outer 10 folds was applied to estimate the classification accuracy, and the inner five folds to select the optimal set of connection features, as was done in previous studies (Whelan et al., 2014; Cui et al., 2016). At the inner fold, 72% (80% of 90%) and 18% (20% of 90%) of individuals were used as training and test sets, respectively. The outer fold for validating the classification model generated a total of 10 models using 90% and 10% of individuals as training and test sets, respectively (Figure 1B). The selection of optimized features was followed using only the training data to avoid circularity (Janssen et al., 2018). A wrapper approach using greedy forward selection was employed to select a subset of features that had the highest relevance to the classification of subtypes (Mwangi et al., 2014).

Logistic regression with ridge estimators (Le Cessie and Van Houwelingen, 1992) was applied as a classification model to identify the individuals’ subtype. Preprocessed connection features were entered into a one-vs.-one algorithm, with the neutral subtype as reference. All procedures of feature selection and model construction with nested cross-validation was conducted using the open-source Waikato Environment for Knowledge Analysis (WEKA) software version 3.8^4^.

Model performance was estimated using the receiver operating characteristics (ROC) analysis. The mean area under the curve (AUC) was calculated for each classification model.

#### Discriminative Features for Each Model

The frequency of the selected features in each fold was calculated to estimate feature contribution and importance in the classification process to identify discriminative connection features for each model. The model performance using logistic regression with ridge estimators of the top 30% of the most frequently selected connection features was estimated with 10-fold cross-validation. In addition, a linear support vector machine classifier was also applied in order to validate the results, as done in a previous study (Cui et al., 2016). The error range for the AUC was computed using a bootstrapping procedure with resampling of 5,000 times. Z-statistics was used to determine whether the AUC is significantly greater than chance (Hanley and McNeil, 1983). The schematic overview of the validation is shown in Figure 1B.

## Results

### Identified Subtypes According to Behavioral and Personality Characteristics

The unsupervised clustering algorithm identified four subtypes, which included the neutral subtype (*n* = 128, 27.0%), A subtype (*n* = 87, 18.3%), B subtype (*n* = 152, 32.0%), and C subtype (*n* = 108, 22.7%; Figure 2A). The detailed clinical characteristics of the participants clustered into each subtype label are summarized in Table 1.

**Table 1:** Behavioral and personality characteristics of each subtype categorized by unsupervised clustering analysis.

	Neutral subtype	A subtype	B subtype	C subtype
No. of individuals	128	87	152	108
Problem drinking, No (%)	3 (2.3)	38 (43.7)	72 (47.4)	5 (4.6)
Behavioral characteristics				
AUDIT scores, mean (SD)	5.01 (3.73)	11.49 (4.83)^a^	12.11 (6.44)^a^	5.53 (3.51)
BIS scores, mean (SD)	59.0 (5.9)	67.7 (5.1)^a^	67.8 (7.8)^a^	66.3 (5.1)^a^
STAXI scores, mean (SD)	23.8 (3.3)	23.7 (2.9)	28.3 (5.5)^a^	30.7 (5.2)^a^
Behavioral approach system scores, mean (SD)	37.3 (4.3)	34.3 (2.2)^a^	41.7 (3.9)^a^	35.6 (4.0)^a^
Behavioral inhibition system scores, mean (SD)	16.5 (3.2)	17.1 (1.8)	19.0 (2.6)^a^	20.6 (3.1)^a^
Personality characteristics				
Extroverted personality dimension^b^, mean (SD)	0.0 (0.69)	0.01 (0.84)	0.94 (0.97)^a^	0.57 (0.92)^a^
Anxious-depressive personality dimension^c^, mean (SD)	0.0 (0.73)	−0.02 (0.77)	0.75 (1.09)^a^	1.15 (1.25)^a^

*^a^The statistical significance at *P* < 0.01 in behavioral and personality characteristics of each subtype as compared with those of the neutral subtype. The general linear models were applied to examine group differences in continuous measures of scale scores after adjusting for sex. ^b^Composite scores for extroverted personality dimensions were calculated by averaging the standardized scores of the subscales for antisocial, borderline, histrionic, and narcissistic personality domains of the PDQ-4+. ^c^Composite scores for anxious-depressive personality dimensions were calculated by averaging the standardized subscale scores of the avoidant, depressive, obsessive-compulsive, and dependent personality domains. No, number; AUDIT, Alcohol Use Disorder Identification Test; BIS, Barratt Impulsiveness Scale; STAXI, State-Trait Anger Expression Inventory; SD, standard deviation*.

The A subtype showed notably higher scores on the BIS (Barratt, 1994; Patton et al., 1995), as compared to the neutral subtype. The B subtype was characterized by high scores on the Behavioral Approach System scale for reward sensitivity, relative to the neutral subtype as well as the other two subtypes. Individuals classified under the B subtype also showed higher scores on other behavioral characteristics including impulsivity, anger, and avoidance. The C subtype displayed a similar profile in behavioral characteristics to the B subtype except for scale scores on the Behavioral Approach System scale.

Scores for the AUDIT (Saunders et al., 1993) were significantly higher in the A subtype (*z* = 10.2, *P* < 0.001) and B subtype (*z* = 11.3, *P* < 0.001) relative to the neutral subtype. However, there were no differences in AUDIT scores between individuals in the C subtype and neutral subtype (*z* = 1.80, *P* = 0.07). Similar to the neutral subtype, the C subtype was the group without any indicators of alcohol misuse. As for the frequency of problem drinking, individuals under the A subtype (*n* = 38, 43.7%) and B subtype (*n* = 72, 47.4%) demonstrated greater potential for problem drinking, which is defined as a score of 12 or higher on the AUDIT (Lee et al., 2000; Figure 2), compared to the neutral (*n* = 3, 2.3%) and C subtypes (*n* = 5, 4.6%). As auxiliary analyses, we have repeated the agglomerative hierarchical clustering analysis using four scale scores, excluding the AUDIT, and a 3-cluster solution was produced. As compared with the original findings, individuals under the C subtype were likely to be combined with the neutral subtype when the AUDIT scores were excluded. Similar to the original findings, there were significant differences in self-reported alcohol use between individuals under the neutral and A subtypes as well as the neutral and B subtypes. Details for these auxiliary analyses are presented in Supplementary Result 1.

In addition, the B subtype (extroverted, *z* = 9.20, *P* < 0.001; anxious-depressive, *z* = 6.63, *P* < 0.001) and C subtype (extroverted, *z* = 4.95, *P* < 0.001; anxious-depressive, *z* = 8.44, *P* < 0.001) revealed higher composite scores on the PDQ-4+ compared to the neutral subtype (Figure 2). There were no differences in composite scores for the two personality dimensions between individuals under the A subtype and neutral subtype (extroverted, *z* = 0.77, *P* = 0.44; anxious-depressive, *z* = 0.04, *P* = 0.97; Figure 2).

### Subtype-Specific Differences in Structural Connections Among the Brain Regions of Addiction Circuitry

Individuals under the A subtype showed significant differences in normalized degrees of the right OFC region (*z* = 2.46, permutation-adjusted *P* = 0.01), right limbic region (*z* = 2.31, permutation-adjusted *P* = 0.02), right interoceptive salience processing region (*z* = −2.39, permutation-adjusted *P* = 0.01), and left interoceptive salience processing region (*z* = −2.07, permutation-adjusted *P* = 0.04) compared to the neutral subtype (Figure 3). Individuals under the C subtype also revealed significant differences in normalized degrees of the right DLPFC region (*z* = 2.27, permutation-adjusted *P* = 0.02), as compared with the neutral subtype (Figure 3). Furthermore, although the differences did not reach statistical significance, the normalized degrees of the left limbic regions were reduced in individuals under the B subtype (*z* = −1.72, permutation-adjusted *P* = 0.09) and C subtype (*z* = −1.77, permutation-adjusted *P* = 0.07) relative to the neutral subtype, respectively. In addition, the normalized degrees of each NOI were also compared between subtypes and presented in Supplementary Table S4.

### Classification of Identified Subtypes According to Neuroimaging Data

**Figure 4:**
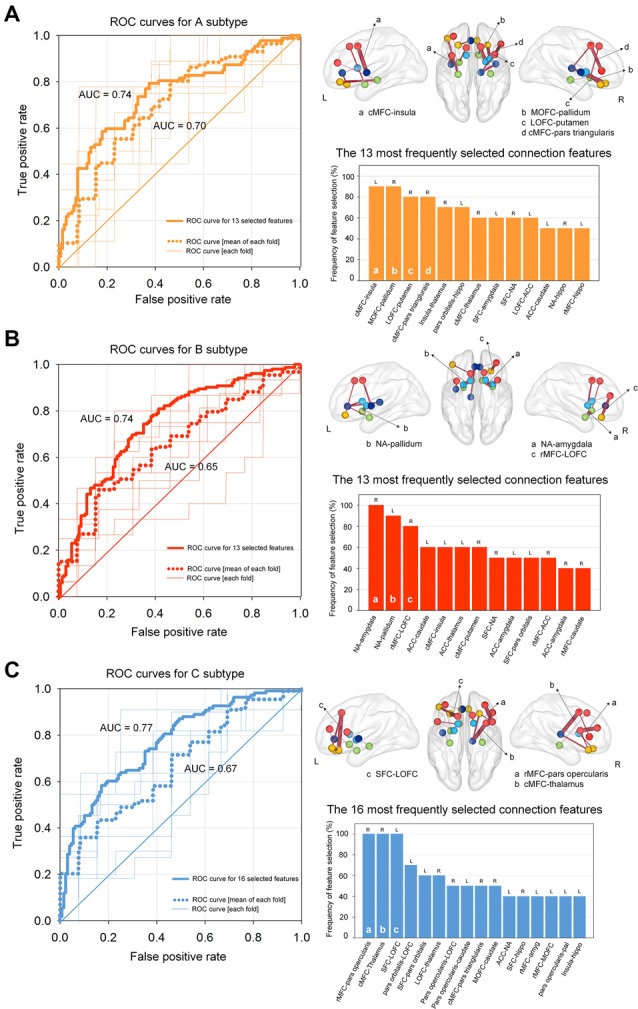
ROC curves for classifying each subtype using regularized logistic regression based on structural connectivity data of the addiction circuitry: **(A)** neutral vs. A subtypes; **(B)** neutral vs. B subtypes; **(C)** neutral vs. C subtypes. Thin lines within each ROC graph indicate the ROC curve from each outer fold test set and dotted lines were obtained by averaging ROC curves of outer 10-fold test sets. Thick lines of the ROC graphs represent the ROC curve from the model based on the most frequently selected connection features for each model. The location and frequency of the most frequently selected structural connections in all successive cross-validation trials were presented in grass brains and bar graphs, respectively. The top 30% of the most frequently selected connection features are presented. ROC, receiver operating characteristic; AUC, area under the curve; cMFC, caudal medial frontal cortex; MOFC, medial orbitofrontal cortex; LOFC, lateral orbitofrontal cortex; NA, nucleus accumbens; ACC, anterior cingulate cortex; SFC, superior frontal cortex; rMFC, rostral medial frontal cortex; L, left; R, right.

The logistic regression analysis with ridge estimators generated three independent binary classification models (neutral vs. A subtypes; neutral vs. B subtypes; neutral vs. C subtypes) using the structural network connection strength within the addiction circuitry as relevant features. Classification performance of three models which identify each subtype relative to the neutral subtype, respectively, yielded the average (overall test sets) AUCs of 0.70, 0.65, and 0.67 for the A subtype, B subtype, and C subtype as compared with the neutral subtype, respectively (Figure 4). We have repeated all analyses using the initially selected feature sets with various selection criteria for the feature reduction step, and the results remained similar (Supplementary Result 2 and Supplementary Figure S1).

The contribution of each connection feature to the classification performance was examined by calculating the frequency of the selected features appearing in cross-validation runs. Frequently selected features were considered to indicate higher reliability, where they provide relevant information for the classification of target subtypes in a consistent manner. The location and frequency of the selected neural connections that best separated each subtype from the neutral subtype and are above the 30th percentile are shown in Figure 4. Discriminative connection features specifically for identifying the A subtype mainly derived from the OFC and dorsal striatal regions. Connections to the limbic regions were robust classifiers for the B subtype, and connections to the DLPFC primarily contributed to the classification of the C subtype relative to the neutral subtype. The list of all the connection features that were selected above a frequency of 0.4 over the repeated cross-validation trials is presented in Supplementary Table S5.

Classification models using only the top 30% of the most frequently selected connection features were also generated, which identified the A subtype, B subtype, and C subtype based on 13, 13 and 16 of the most relevant connection features, respectively. Individuals classified under the A subtype, B subtype, and C subtype were discriminated from those under the neutral subtype with AUCs of 0.74 [95% confidence interval (CI) = 0.67–0.81, *P* < 0.001], 0.74 (95% CI = 0.66 -0.82, *P* < 0.001), and 0.77 (95% CI = 0.70–0.84, *P* < 0.001), respectively (Figure 4). Histograms of AUCs after bootstrapping procedure with resampling of 5,000 times are presented in Supplementary Figure S1, where the dashed black lines indicate the AUC for classifying each subtype apart from the neutral subtype. To validate the robustness of the current results, we additionally applied the linear support vector machine classifier, which is similar and comparable to the regularized logistic regression in obtaining a linear classifier (Cui et al., 2016), for classifying the three subtypes relative to the neutral subtype, respectively. Specifically, the linear support vector machine models used the same feature sets that included the 13, 13, and 16 most relevant connection features to classify A, B, and C subtypes, respectively, from the neutral subtype. The accuracy of the AUCs in classifying the A, B, and C subtypes were 0.73 (95% CI = 0.64–0.81, *P* < 0.001), 0.73 (95% CI = 0.65–0.80, *P* < 0.001), and 0.75 (95% CI = 0.68–0.83, *P* < 0.001), respectively. Histograms of the AUCs using bootstrapping resampling (*n* = 5,000) are presented in Supplementary Figure S2.

## Discussion

The present study identified neurobiologically distinct subtypes in young adults without any prior history of addictive disorders and examined whether patterns of alcohol misuse may differ according to the subtypes. Specifically, the current sample of young adults has been classified into four subtypes, each of which demonstrated unique behavioral tendency and differential propensity to alcohol misuse. Each of the four identified subtypes was then further validated and supported through neurobiological data, by their unique patterns of structural connections among brain regions within the addiction circuitry.

With the neutral subtype as the reference point, the three other subtypes exhibited significantly distinct type-specific behavioral and personality characteristics. For instance, approximately 18% of the participants were characterized as having an impulsive tendency (A subtype), while those who exhibited high levels of behavioral symptoms related to addictive behaviors, such as reward sensitivity, anger, impulsivity, and avoidance were defined as the B subtype. Individuals under the A subtype as well as B subtype were more likely to show problematic drinking patterns as compared with those under the neutral subtype, indicating a potential risk for an addictive disorder. This finding is partly consistent with previous reports which suggest the predictive roles of impulsivity and reward sensitivity in vulnerability to addiction (Castellanos-Ryan et al., 2013; Egervari et al., 2018). Furthermore, several preclinical studies also indicated that impaired impulse control plays a central role in the progression to addiction (Fineberg et al., 2010; Winstanley et al., 2010; Ersche et al., 2012a,b; Jupp et al., 2013). Specifically, impulsivity may contribute to future drug self-administration and drug exposure, along with increased impulsive responsiveness (de Wit, 2009; Winstanley et al., 2010). The C subtype consisted of 23% of the participants and was characterized as more avoidant, impulsive, and angry, as compared with the neutral subtype. However, the scores related to reward-sensitivity within the C subtype were similar to those under the neutral subtype. In addition, individuals within the C subtype were less likely to engage in alcohol misuse, compared to the other A and B subtypes. Considering that the major distinction in terms of behavioral traits between individuals under the B and C subtypes was leveled on reward sensitivity, the contrasting traits between the two subtypes may indicate that differences in responsiveness to rewards may lower the risk for alcohol misuse.

More importantly, the current study identified potential neurobiological characteristics that were most relevant to the discrimination of subtypes. In particular, our cross-validation approach demonstrated that the connections to the OFC and limbic regions may play an important role in distinguishing the A subtype apart from the neutral subtype. The current findings support previous evidence in that the OFC and limbic structure have been identified as important regions for impulse control (Winstanley, 2007), and functional and structural alterations in the OFC are evident in individuals with substance and alcohol abuse (Crews and Boettiger, 2009; Moorman, 2018). Given that the OFC and its connected regions are also considered as common neural circuitry that underlies impulsivity and addictive behavior (Volkow and Fowler, 2000; Winstanley, 2007; Volkow and Morales, 2015), altered connections to the OFC may contribute to the behavioral manifestations of impulsive tendency such as comorbid alcohol misuse.

Furthermore, the current results indicate that alteration in connections of the limbic regions including the nucleus accumbens is robust classifiers for distinguishing between individuals under the B subtype and neutral subtype. Notably, individuals under the B subtype also exhibited higher levels of propensity to other risk factors of addiction including impulsivity, anger, and avoidance. The interaction between the nucleus accumbens, limbic system and striatum has been known to modulate motivational behavior, and play a significant role in adolescent or early adult-onset addictive disorder (Sharma and Morrow, 2016; Volkow et al., 2016). Also, this behavioral constellation of the B subtype as observed in the present study has been reported to be highly prevalent in early-onset alcohol abusers (Dom et al., 2006) and associated with an increased risk for clinically relevant addictive disorders later in life (Dom et al., 2006; Nees et al., 2012).

The C subtype demonstrated higher scores on the behavioral inhibition system while having lower scores on reward sensitivity. Individuals under the C subtype also exhibited high levels of anger and impulsivity, compared to the neutral subtype. Given that problem drinking were less frequent in this subtype, individuals clustered to the C subtype may refrain from particular risky behaviors in order to prevent facing negative consequences. Here, we speculate that increased connections to the DLPFC within the addiction circuitry as found in young adults under this subtype may contribute to a more efficient control over risk-taking behaviors such as impulsivity and anger. In addition, high levels of impulsivity shown in individuals under the C subtype seem efficiently controlled and may be able to refrain from the manifestation of risky behaviors such as excessive alcohol consumption. The C subtype demonstrated heightened connections of the DLPFC within the addictive circuitry, which may indicate the potential role of the DLPFC and its connections to other addiction-related brain regions in controlling impulsivity.

Limitation of the present study should also be discussed. While the addiction circuitry has become a familiarized topic in addiction research, debates continue regarding regions that make up the addiction circuitry. Considering the sufficient evidence regarding cerebellar alterations in many comorbid neuropsychiatric disorders including addiction (Miquel et al., 2016), future studies could extend the current findings with the inclusion of additional brain regions that are significant to addictive behaviors. Moreover, independent replication of the current findings in a clinically-diagnosed sample of addictive disorders may obtain adequate generalizability of the current model. It is also noteworthy that altered patterns of brain regional connectivity caused by chronic substance use would differ from those underlying the vulnerability towards substance use disorders (Ersche et al., 2012a,b; Schulte et al., 2012; Whelan et al., 2014). Future longitudinal studies on individuals who are at high risk for developing addictive disorders is warranted to discriminate between the brain network correlates of vulnerability to addiction and those of adaptive or neurodegenerative changes due to chronic addiction.

The current study also corrected for eddy current using the “eddy_correct” function implemented in the FSL toolbox and used the FACT algorithm to reconstruct white matter fiber tracts. While the current approaches are acceptable ways to preprocess image data, future DTI studies using more up-to-date preprocessing methodologies such as enabling of both eddy current- and susceptibility-induced distortion corrections (Sotiropoulos et al., 2013; Yamada et al., 2014), as well as fiber tracking by probabilistic tractography algorithm (Li et al., 2013; Jenabi et al., 2015), may outperform the current results.

Furthermore, despite excluding individuals with a past diagnosis of alcohol or substance dependence, the current sample may include those who refrain or abstain from drinking alcohol due to previous adverse reactions to alcohol use (Hughes et al., 1985; Stockwell et al., 2016). Considering that deliberate effort to refrain from alcohol such as in individuals who practice teetotalism may influence individual traits such as mood (Randall et al., 2004), future studies that distinguish individuals who refrain from alcohol use due to miscellaneous justifications may provide supportive evidence in the distinct personality and behavioral traits in relation to alcohol misuse. The mean age of the current sample (27.1 years old) may also be higher than the typical age of initiation for addictive behaviors (Grant et al., 2001; Pitkänen et al., 2005; Pilatti et al., 2017). Therefore, the present findings should be interpreted with caution, and further research that replicates these findings using a longitudinal approach may provide additional robust evidence for a causal link between personality traits and problematic alcohol use. Nevertheless, we were able to predict problematic alcohol use measured using the AUDIT from personality characteristics, showing the potential of machine learning in precision psychiatry.

In summary, we identified distinct subtypes related to alcohol misuse that are both theoretically plausible and neurobiologically supportive in a group of young adults using a data-driven approach that incorporates their behavioral and personality characteristics. The high classification accuracy of the identified subtypes according to brain connection patterns may further validate that the current classification model of subtypes has significant biological relevance. Our findings, if replicated longitudinally, may contribute to the development of models that better predict individual vulnerability to the future onset of devastating addictive disorders, including the alcohol and other substance dependence, in healthy young adults. This approach may then further aid in the development of targeted, personalized strategies of prevention and intervention for addictive disorders according to individual behavioral and personality characteristics.

## Data Availability Statement

The datasets generated for this study are available on request to the corresponding author.

## Ethics Statement

The studies involving human participants were reviewed and approved by the Institutional Review Board of Ewha W. University. The patients/participants provided their written informed consent to participate in this study.

## Author Contributions

SY and IL designed the study. SY, JK, GH, TK, HH, EH, JM, and IL acquired the data, which SY, JK, GH, JM, and IL analyzed. SY, JK, TK, and IL wrote the manuscript, which all authors reviewed and edited. All authors approved the final version to be published and can certify that no other individuals not listed as authors have made substantial contributions to the manuscript.

## Conflict of Interest

The authors declare that the research was conducted in the absence of any commercial or financial relationships that could be construed as a potential conflict of interest.

## Footnotes

^1^http://trackvis.org/dtk

^2^http://trackvis.org

^3^http://surfer.nmr.mgh.harvard.edu

^4^https://www.cs.waikato.ac.nz/ml/weka/

## References

[B1] Barkin S. L. Smith K. S. DuRant R. H. (2002). Social skills and attitudes associated with substance use behaviors among young adolescents. J. Adolesc. Health 30, 448–454. 10.1016/s1054-139x(01)00405-0 12039515

[B2] Barratt E. S. (1994). “Impulsiveness and aggression,” in Violence and Mental Disorder: Developments in Risk Assessment, eds Monahan J. Steadman H. J. (Chicago, IL: University of Chicago Press), 61–79.

[B3] Belcher A. M. Volkow N. D. Moeller F. G. Ferré S. (2014). Personality traits and vulnerability or resilience to substance use disorders. J. Dual Diagn. 18, 211–217. 10.1016/j.tics.2014.01.010 24612993 PMC3972619

[B4] Berglund K. Roman E. Balldin J. Berggren U. Eriksson M. Gustavsson P.. (2011). Do men with excessive alcohol consumption and social stability have an addictive personality? Scand. J. Psychol. 52, 257–260. 10.1111/j.1467-9450.2010.00872.x 21255023

[B5] Carver C. S. White T. L. (1994). Behavioral inhibition, behavioral activation and affective responses to impending reward and punishment: the BIS/BAS scales. J. Pers. Soc. Psychol. 67, 319–333. 10.1037/0022-3514.67.2.319

[B6] Castellanos-Ryan N. O’Leary-Barrett M. Sully L. Conrod P. (2013). Sensitivity and specificity of a brief personality screening instrument in predicting future substance use, emotional and behavioral problems: 18-month predictive validity of the substance use risk profile scale. Alcohol. Clin. Exp. Res. 37, E281–E290. 10.1111/j.1530-0277.2012.01931.x 22974180

[B7] Crews F. T. Boettiger C. A. (2009). Impulsivity, frontal lobes and risk for addiction. Pharmacol. Biochem. Behav. 93, 237–247. 10.1016/j.pbb.2009.04.018 19410598 PMC2730661

[B8] Cui Z. Xia Z. Su M. Shu H. Gong G. (2016). Disrupted white matter connectivity underlying developmental dyslexia: a machine learning approach. Hum. Brain Mapp. 37, 1443–1458. 10.1002/hbm.23112 26787263 PMC6867308

[B10] Deleuze J. Rochat L. Romo L. Van der Linden M. Achab S. Thorens G.. (2015). Prevalence and characteristics of addictive behaviors in a community sample: a latent class analysis. Addict. Behav. Rep. 1, 49–56. 10.1016/j.abrep.2015.04.001 29531979 PMC5845955

[B11] Desikan R. S. Segonne F. Fischl B. Quinn B. T. Dickerson B. C. Blacker D.. (2006). An automated labeling system for subdividing the human cerebral cortex on MRI scans into gyral based regions of interest. NeuroImage 31, 968–980. 10.1016/j.neuroimage.2006.01.021 16530430

[B9] de Wit H. (2009). Impulsivity as a determinant and consequence of drug use: a review of underlying processes. Addict. Biol. 14, 22–31. 10.1111/j.1369-1600.2008.00129.x 18855805 PMC3640851

[B12] Dom G. Hulstijn W. Sabbe B. (2006). Differences in impulsivity and sensation seeking between early- and late-onset alcoholics. Addict. Behav. 31, 298–308. 10.1016/j.addbeh.2005.05.009 15949898

[B13] Egervari G. Ciccocioppo R. Jentsch J. D. Hurd Y. L. (2018). Shaping vulnerability to addiction - the contribution of behavior, neural circuits and molecular mechanisms. Neurosci. Biobehav. Rev. 85, 117–125. 10.1016/j.neubiorev.2017.05.019 28571877 PMC5708151

[B14] Ersche K. D. Jones P. S. Williams G. B. Smith D. G. Bullmore E. T. Robbins T. W. (2013). Distinctive personality traits and neural correlates associated with stimulant drug use versus familial risk of stimulant dependence. Biol. Psychiatry 74, 137–144. 10.1016/j.biopsych.2012.11.016 23273722 PMC3705207

[B15] Ersche K. D. Jones P. S. Williams G. B. Turton A. J. Robbins T. W. Bullmore E. T. (2012a). Abnormal brain structure implicated in stimulant drug addiction. Science 335, 601–604. 10.1126/science.1214463 22301321

[B16] Ersche K. D. Turton A. J. Chamberlain S. R. Müller U. Bullmore E. T. Robbins T. W. (2012b). Cognitive dysfunction and anxious-impulsive personality traits are endophenotypes for drug dependence. Am. J. Psychiatry 169, 926–936. 10.1176/appi.ajp.2012.11091421 22952072 PMC3533378

[B17] Fineberg N. A. Potenza M. N. Chamberlain S. R. Berlin H. A. Menzies L. Bechara A.. (2010). Probing compulsive and impulsive behaviors, from animal models to endophenotypes: a narrative review. Neuropsychopharmacology 35, 591–604. 10.1038/npp.2009.185 19940844 PMC3055606

[B18] First M. B. Spitzer R. L. Gibbon M. Williams J. B. W. (1997). Structured Clinical Interview for DSM-IV Axis I Disorders (SCID I), Clinical Version. Washington, DC: American Psychiatric Press.

[B19] Friedman J. Hastie T. Tibshirani R. (2001). The Elements of Statistical Learning. New York, NY: Springer Series in Statistics.

[B20] Gabrieli J. D. E. Ghosh S. S. Whitfield-Gabrieli S. (2015). Prediction as a humanitarian and pragmatic contribution from human cognitive neuroscience. Neuron 85, 11–26. 10.1016/j.neuron.2014.10.047 25569345 PMC4287988

[B21] Grant B. F. Stinson F. S. Harford T. C. (2001). Age at onset of alcohol use and DSM-IV alcohol abuse and dependence: a 12-year follow-up. J. Subst. Abuse 13, 493–504. 10.1016/s0899-3289(01)00096-7 11775078

[B22] Hanley J. A. McNeil B. J. (1983). A method of comparing the areas under receiver operating characteristic curves derived from the same cases. Radiology 148, 839–843. 10.1148/radiology.148.3.6878708 6878708

[B23] Heinrich A. Müller K. U. Banaschewski T. Barker G. J. Bokde A. L. Bromberg U.. (2016). Prediction of alcohol drinking in adolescents: personality-traits, behavior, brain responses and genetic variations in the context of reward sensitivity. Biol. Psychol. 118, 79–87. 10.1016/j.biopsycho.2016.05.002 27180911

[B24] Helfinstein S. M. Schonberg T. Congdon E. Karlsgodt K. H. Mumford J. A. Sabb F. W.. (2014). Predicting risky choices from brain activity patterns. Proc. Natl. Acad. Sci. U S A 111, 2470–2475. 10.1073/pnas.1321728111 24550270 PMC3932884

[B25] Hughes J. Stewart M. Barraclough B. (1985). Why teetotallers abstain. Br. J. Psychiatry 146, 204–206. 10.1192/bjp.146.2.204 3978336

[B26] Hyler S. E. (1994). Personality Diagnostic Questionnaire-4+. New York: New York State Psychiatric Institute.

[B27] Janssen R. J. Mourão-Miranda J. Schnack H. G. (2018). Making individual prognoses in psychiatry using neuroimaging and machine learning. Biol. Psychiatry Cogn. Neurosci. Neuroimaging 3, 798–808. 10.1016/j.bpsc.2018.04.004 29789268

[B28] Jenabi M. Peck K. K. Young R. J. Brennan N. Holodny A. I. (2015). Identification of the corticobulbar tracts of the tongue and face using deterministic and probabilistic DTI fiber tracking in patients with brain tumor. Am. J. Neuroradiol. 36, 2036–2041. 10.3174/ajnr.a4430 26251424 PMC4982871

[B29] Jupp B. Caprioli D. Dalley J. W. (2013). Highly impulsive rats: modelling an endophenotype to determine the neurobiological, genetic and environmental mechanisms of addiction. Dis. Model. Mech. 6, 302–311. 10.1242/dmm.010934 23355644 PMC3597013

[B30] Koob G. F. Volkow N. D. (2016). Neurobiology of addiction: a neurocircuitry analysis. Lancet Psychiatry 3, 760–773. 10.1016/S2215-0366(16)00104-8 27475769 PMC6135092

[B31] Le Cessie S. Van Houwelingen J. C. (1992). Ridge estimators in logistic regression. J. R. Stat. Soc. Ser. C 41, 191–201.

[B32] Lee B. O. Lee C. H. Lee P. G. Choi M. J. Namkoong K. (2000). Development of Korean version of alcohol use disorders identification test (AUDIT-K): its reliability and validity. J. Korean Acad. Addict. Psychiatry 4, 83–92.

[B33] Li Z. Peck K. K. Brennan N. P. Jenabi M. Hsu M. Zhang Z.. (2013). Diffusion tensor tractography of the arcuate fasciculus in patients with brain tumors: comparison between deterministic and probabilistic models. J. Biomed. Sci. Eng. 6, 192–200. 10.4236/jbise.2013.62023 25328583 PMC4199232

[B34] Lim K. O. Choi S. J. Pomara N. Wolkin A. Rotrosen J. P. (2002). Reduced frontal white matter integrity in cocaine dependence: a controlled diffusion tensor imaging study. Biol. Psychiatry 51, 890–895. 10.1016/s0006-3223(01)01355-5 12022962

[B35] Miquel M. Vazquez-Sanroman D. Carbo-Gas M. Gil-Miravet I. Sanchis-Segura C. Carulli D.. (2016). Have we been ignoring the elephant in the room? Seven argument for considering the cerebellum as part of addiction circuitry. Neurosci. Biobehav. Rev. 60, 1–11. 10.1016/j.neubiorev.2015.11.005 26602022

[B36] Moorman D. E. (2018). The role of the orbitofrontal cortex in alcohol use, abuse and dependence. Prog. Neuropsychopharmacol. Biol. Psychiatry 87, 85–107. 10.1016/j.pnpbp.2018.01.010 29355587 PMC6072631

[B37] Mori S. Crain B. J. Chacko V. P. Van Zijl P. C. (1999). Three-dimensional tracking of axonal projections in the brain by magnetic resonance imaging. Ann. Neurol. 45, 265–269. 10.1002/1531-8249(199902)45:2<265::aid-ana21>3.0.co;2-3 9989633

[B38] Mwangi B. Tian T. S. Soares J. C. (2014). A review of feature reduction techniques in neuroimaging. Neuroinformatics 12, 229–244. 10.1007/s12021-013-9204-3 24013948 PMC4040248

[B39] Nees F. Tzschoppe J. Patrick C. J. Vollstädt-Klein S. Steiner S. Poustka L.. (2012). Determinants of early alcohol use in healthy adolescents: the differential contribution of neuroimaging and psychological factors. Neuropsychopharmacology 37, 986–995. 10.1038/npp.2011.282 22113088 PMC3280646

[B40] Nigg J. T. Wong M. M. Martel M. M. Jester J. M. Puttler L. I. Glass J. M.. (2006). Poor response inhibition as a predictor of problem drinking and illicit drug use in adolescents at risk for alcoholism and other substance use disorders. J. Am. Acad. Child Adolesc. Psychiatry 45, 468–475. 10.1097/01.chi.0000199028.76452.a9 16601652

[B41] Patton J. H. Stanford M. S. Barratt E. S. (1995). Factor structure of the Barratt impulsiveness scale. J. Clin. Psychol 51, 768–774. 10.1002/1097-4679(199511)51:6<768::aid-jclp2270510607>3.0.co;2-1 8778124

[B42] Pilatti A. Read J. P. Pautassi R. M. (2017). ELSA 2016 cohort: alcohol, tobacco and marijuana use and their association with age of drug use onset, risk perception and social norms in Argentinean college freshmen. Front. Psychol. 8:1452. 10.3389/fpsyg.2017.01452 28890707 PMC5575425

[B43] Pitkänen T. Lyyra A. L. Pulkkinen L. (2005). Age of onset of drinking and the use of alcohol in adulthood: a follow-up study from age 8–42 for females and males. Addiction 100, 652–661. 10.1111/j.1360-0443.2005.01053.x 15847623

[B44] Poldrack R. A. Monahan J. Imrey P. B. Reyna V. Raichle M. E. Faigman D.. (2018). Predicting violent behavior: what can neuroscience add? Trends Cogn. Sci. 22, 111–123. 10.1016/j.tics.2017.11.003 29183655 PMC5794654

[B45] Randall D. C. Elsabagh S. M. Hartley D. E. File S. E. (2004). Does drinking have effects on mood and cognition in male and female students? Pharmacol. Biochem. Behav. 78, 629–638. 10.1016/j.pbb.2004.04.029 15251272

[B46] Romero M. J. Asensio S. Palau C. Sanchez A. Romero F. J. (2010). Cocaine addiction: diffusion tensor imaging study of the inferior frontal and anterior cingulate white matter. Psychiatry Res. 181, 57–63. 10.1016/j.pscychresns.2009.07.004 19959341

[B47] Rubinov M. Sporns O. (2010). Complex network measures of brain connectivity: uses and interpretations. NeuroImage 52, 1059–1069. 10.1016/j.neuroimage.2009.10.003 19819337

[B48] Saunders J. B. Aasland O. G. Babor T. F. de la Fuente J. R. Grant M. (1993). Development of the alcohol use disorders identification test (AUDIT): WHO collaborative project on early detection of persons with harmful alcohol consumption-II. Addiction 88, 791–804. 10.1111/j.1360-0443.1993.tb02093.x 8329970

[B49] Schulte T. Oberlin B. G. Kareken D. A. Marinkovic K. Müller-Oehring E. M. Meyerhoff D. J.. (2012). How acute and chronic alcohol consumption affects brain networks: insights from multimodal neuroimaging. Alcohol. Clin. Exp. Res. 36, 2017–2027. 10.1111/j.1530-0277.2012.01831.x 22577873 PMC4500115

[B50] Sharma A. Morrow J. D. (2016). Neurobiology of adolescent substance use disorders. Child Adolesc. Psychiatr. Clin. N. Am. 25, 367–375. 10.1016/j.chc.2016.02.001 27338961

[B51] Sotiropoulos S. N. Jbabdi S. Xu J. Andersson J. L. Moeller S. Auerbach E. J.. (2013). Advances in diffusion MRI acquisition and processing in the human connectome project. NeuroImage 80, 125–143. 10.1016/j.neuroimage.2013.05.057 23702418 PMC3720790

[B52] Spielberger C. D. (1988). Manual for the State-Trait Anger Expression Inventory (STAXI). Odessa, FL: Psychological Assessment Resources.

[B53] Stockwell T. Zhao J. Panwar S. Roemer A. Naimi T. Chikritzhs T. (2016). Do “Moderate” drinkers have reduced mortality risk? A systematic review and meta-analysis of alcohol consumption and all-cause mortality. J. Stud. Alcohol Drugs 77, 185–198. 10.15288/jsad.2016.77.185 26997174 PMC4803651

[B54] Tarter R. E. Kirisci L. Mezzich A. Cornelius J. R. Pajer K. Vanyukov M.. (2003). Neurobehavioral disinhibition in childhood predicts early age at onset of substance use disorder. Am. J. Psychiatry 160, 1078–1085. 10.1176/appi.ajp.160.6.1078 12777265

[B55] Terracciano A. Löckenhoff C. E. Crum R. M. Bienvenu O. J. Costa P. T. (2008). Five-factor model personality profiles of drug users. BMC Psychiatry 8:22. 10.1186/1471-244X-8-22 18405382 PMC2373294

[B56] Volkow N. D. Fowler J. S. (2000). Addiction, a disease of compulsion and drive: involvement of the orbitofrontal cortex. Cereb. Cortex 10, 318–325. 10.1093/cercor/10.3.318 10731226

[B58] Volkow N. D. Koob G. F. McLellan A. T. (2016). Neurobiologic advances from the brain disease model of addiction. N. Engl. J. Med 374, 363–371. 10.1056/NEJMra1511480 26816013 PMC6135257

[B57] Volkow N. D. Morales M. (2015). The brain on drugs: from reward to addiction. Cell 162, 712–725. 10.1016/j.cell.2015.07.046 26276628

[B59] Ward J. H. (1963). Hierarchical grouping to optimize an objective function. J. Am. Stat. Assoc. 58, 236–244. 10.1080/01621459.1963.10500845

[B60] Westfall P. H. Young S. S. (1993). Resampling-Based Multiple Testing: Examples and Methods for P-value Adjustment. New York, NY: John Wiley and Sons.

[B61] Whelan R. Watts R. Orr C. A. Althoff R. R. Artiges E. Banaschewski T.. (2014). Neuropsychosocial profiles of current and future adolescent alcohol misusers. Nature 512, 185–189. 10.1038/nature13402 25043041 PMC4486207

[B62] Winstanley C. A. (2007). The orbitofrontal cortex, impulsivity and addiction: probing orbitofrontal dysfunction at the neural, neurochemical and molecular level. Ann. N Y Acad. Sci. 1121, 639–655. 10.1196/annals.1401.024 17846162

[B63] Winstanley C. A. Olausson P. Taylor J. R. Jentsch J. D. (2010). Insight into the relationship between impulsivity and substance abuse from studies using animal models. Alcohol. Clin. Exp. Res. 34, 1306–1318. 10.1111/j.1530-0277.2010.01215.x 20491734 PMC3380443

[B64] Yamada H. Abe O. Shizukuishi T. Kikuta J. Shinozaki T. Dezawa K.. (2014). Efficacy of distortion correction on diffusion imaging: comparison of FSL eddy and eddy_correct using 30 and 60 directions diffusion encoding. PLoS One 9:e112411. 10.1371/journal.pone.0112411 25405472 PMC4236106

[B65] Yoon S. Kim J. E. Hwang J. Kim T. S. Kang H. J. Namgung E.. (2016). Effects of creatine monohydrate augmentation on brain metabolic and network outcome measures in women with major depressive disorder. Biol. Psychiatry 80, 439–447. 10.1016/j.biopsych.2015.11.027 26822799

[B66] Zhang R. Jiang G. Tian J. Qiu Y. Wen X. Zalesky A.. (2016). Abnormal white matter structural networks characterize heroin-dependent individuals: a network analysis. Addict. Biol. 21, 667–678. 10.1111/adb.12234 25740690

