# Supplementary material for: Identification of Tendency to Alcohol Misuse From the Structural Brain Networks
Source: Front Syst Neurosci. 2020 Mar 3;14:9. doi: 10.3389/fnsys.2020.00009 (PMC7062673; doi:10.3389/fnsys.2020.00009)
Supplement: Supplementary file 1 [file Data_Sheet_1.docx]

Supplementary Material

**Supplementary Methods**

**Behavioral and personality assessments**

***Alcohol Use Disorder Identification Test (AUDIT)***

The AUDIT (Saunders *et al*., 1993) is a screening tool to assess alcohol consumption, drinking behaviors, and alcohol-related problems. The self-report version of the AUDIT is a 10-item questionnaire which has been validated across gender as well as various racial/ethnic groups. Total scores of the AUDIT were used as a measure for alcohol-related problems with a cut-off score of 12 to define problem drinking (Lee *et al*., 2000).

***Barratt Impulsiveness Scale (BIS)***

The BIS (Barratt, 1994; Patton *et al*., 1995) is a 30-item self-questionnaire for multifaceted measurement of impulsiveness. Each item is scored on a 4-point Likert scale. Three subscales of attentional, motor, and non-planning impulsiveness are used to assess personality and/or behavioral traits related to impulsiveness. Total scores of the three subscales of the BIS were used as a measure for the level of impulsivity.

***State-Trait Anger Expression Inventory (STAXI)***

The STAXI (Spielberger, 1988) is a 44-item self-report questionnaire which consists of 5 independent subscales of anger: State Anger, Trait Anger, Anger-in, Anger-out, and Anger Control. The scale assesses the components of anger in the context of normal and abnormal personality, as well as the potential role of anger in certain medical illnesses and conditions. Each item is rated on a 4-point Likert scale, ranging from ‘almost never’ to ‘almost always’. The combined scores of the State Anger and Trait Anger subscales were used as a measure for the level of anger.

***Behavioral Inhibition System/Behavioral Approach System Scales***

The Behavioral Inhibition System scale is comprised of 7 items each rated on a 4-point Likert scale to assess the avoidance and anxiety responsivity to aversive stimuli (Carver and White, 1994). The Behavioral Approach System Scale consists of 13 items and is subdivided into 3 subscales which measures fun-seeking, reward responsiveness, and drive, respectively. Persons with high scores on the Behavioral Approach System Scale are more likely to experience positive affects in response to reward-associated stimuli and engage in risk-taking behaviors (Carver and White, 1994). The total scale scores for the Behavioral Inhibition System and Behavioral System were used as measures for avoidance/anxiety and reward dependency, respectively.

***Personality Diagnostic Questionnaire-4th Edition Plus (PDQ-4+)***

The PDQ-4+ (Hyler, 1994) is a 99-item self-report questionnaire that measures the 10 personality disorders in DSM-IV (paranoid, schizoid, schizotypal, antisocial, borderline, histrionic, narcissistic, avoidant, dependent, and obsessive-compulsive personality disorders) and 2 additional characteristics including passive-aggressive and depressive personality disorders. In the current study, composite scores for two personality tendencies which are extroverted and anxious-depressive characteristics were calculated to assess the dominant personality characteristics. Composite scores for extroverted personality tendency were calculated by averaging the standardized scores of subscales for antisocial, borderline, histrionic, and narcissistic personality and those for anxious-depressive personality tendency were calculated by averaging the standardized scores of subscales for avoidant, depressive, obsessive-compulsive, and dependent personality.

**Neuroimaging data acquisition and preprocessing**

***Magnetic resonance imaging (MRI) data acquisition***

High-resolution structural and resting-state functional MRI scans were obtained using a 3.0 Tesla Philips Achieva MR scanner (Philips Medical System, Netherlands) equipped with a 32-channel head coil. High-resolution T1-weighted images were acquired using the following acquisition parameters: repetition time (TR), 7.4 ms; echo time (TE), 3.4 ms; flip angle (FA), 8°; field of view (FOV), 220 X 220 mm^2^; slice thickness, 1 mm; 180 contiguous sagittal slices. Thirty-two directions of diffusion-weighted images (b, 1000 s/m^2^; TR, shortest [9,100-9,300 ms]; TE, 68 ms; flip angle, 90°; FOV, 200 X 200 mm^2^; number of excitation, 2; slice thickness, 1.8 mm) and one image without diffusion weighting (b, 0 s/m^2^) were also acquired.

***T1-weighted and diffusion-weighted image preprocessing and tractography***

Seventeen brain regions per each hemisphere were selected as nodes of interest (NOIs) of the addiction circuitry. The list of NOIs is presented in Table S2. For fiber tracking, masks for the NOIs were derived from the high-resolution T1-weighted images of each individual using the FreeSurfer tool (http://surfer.nmr.mgh.harvard.edu). Individual brain were parcellated based on the T1-weighted images into different cortical and subcortical regions per each hemisphere (Desikan *et al*., 2006). Among a set of cortical and subcortical masks, 34 masks were selected for constructing the brain network. For the registration of NOI masks to the diffusion space, non-diffusion b0 images of each individual was coregistered to the respective T1-weighted image using affine transformation. By using the inverse transformation matrix, all sets of NOI masks were transformed to the diffusion space.

All diffusion-weighted images were corrected for head motion or eddy current distortions including eddy current-induced image shearing, translation, or stretching based on affine transformation by using the eddy_correct function implemented in the FSL toolbox (https://fsl.fmrib.ox.ac.uk/fsl/fslwiki), as done in previous studies (Stricker *et al.*, 2009; Rae *et al.*, 2012; Sorg *et al.*, 2012; Wiech *et al.*, 2014; Zorlu *et al.*, 2019). While noting that the current method of head motion or eddy current distortions is an acceptable approach, it is worth considering that the eddy_correct function is unable to correct for susceptibility-induced distortions, unlike the more recently developed pipeline that combined eddy and topup functions simultaneously for eddy current- and susceptibility-induced distortions (Sotiropoulos *et al.*, 2013; Yamada *et al.*, 2014). The Diffusion Toolkit (http://trackvis.org/dtk) was used to calculate diffusion tensor and the Fiber Assignment by Continuous Tracking (FACT) algorithm was applied to reconstruct the white matter fiber tracts interconnecting the 17 NOIs in each hemisphere (Mori et al., 1999) using the Trackvis (http://trackvis.org) software package. Eight streamline seeds were selected for each voxel within the NOI and initialized to follow the main diffusion direction. The stopping criteria for the streamline were as follows: 1) a fiber tract reached a voxel with a fractional anisotropy value lower than 0.1 and 2) the streamline reached an angle greater than 45 degrees or the streamline exceeded the NOI.

**Supplementary Results**

**Supplementary Result 1**

Agglomerative hierarchical clustering analysis was also performed on the standardized scores of the 4 scales including the BIS, STAXI, Behavioral Inhibition System scale and Behavioral Approach System scale. The analysis produced a 3-cluster solution, where the C subtype from the original findings was likely to be combined with the neutral subtype. The 3 clusters included the neutral subtype (n = 171; scale scores, mean ± SD; BIS, 59.6 ± 5.3; STAXI, 23.9 ± 3.4; Behavioral Inhibition System scale, 17.1 ± 3.5; Behavioral Approach System scale, 37.1 ± 4.1), A subtype (n = 167; scale scores, mean ± SD; BIS, 69.2 ± 4.5; STAXI, 27.7 ± 5.2; Behavioral Inhibition System scale, 18.3 ± 2.1; Behavioral Approach System scale, 34.7 ± 3.0), and B subtype (n = 139; scale scores, mean ± SD; BIS, 66.8 ± 7.9; STAXI, 29.6 ± 6.5; Behavioral Inhibition System scale, 19.9 ± 3.2; Behavioral Approach System scale, 42.3 ± 3.7). Similar to the original findings, individuals under the A subtype showed higher levels of impulsivity as compared to the neutral subtype, while individuals under the B subtype had higher scores on all 4 scales scores for impulsivity, anger, avoidance, and reward sensitivity. In addition, significant between-group differences were found in self-reported alcohol use as measured by AUDIT, specifically between the neutral and A subtypes (*z* = 2.55, *P* = 0.01) as well as the neutral and B subtypes (*z* = 2.79, *P* = 0.005).

**Supplementary Result 2**

In the initial feature reduction step, the top 20% of connection features (n = 54) showing group differences as compared to the neutral subtype were selected out of 272 connection features as the input features for each classification model. As sensitivity analyses, we repeated the estimation of the model performance using initially selected feature sets with two distinct selection criteria (top 10%, n = 27; top 30%, n =81). When selecting the top 10% of features with the greatest between-group difference as an initial feature reduction step (n = 27), the averaged (over all test sets) area under the curve (AUC) for classification performance of models that classified the A, B, and C subtypes relative to the neutral subtype were 0.69, 0.70, and 0.74, respectively. Classification models were also generated using the initially selected features according to top the 30% of features with the greatest between-group difference as an initial feature reduction step (n = 81), where the averaged AUC for classification models predicting the A, B, and C subtypes, relative to the neutral subtype were 0.60, 0.66, and 0.64, respectively. ROC curves for these classification models with different selection criteria for the initial feature reduction step are also presented in Figure S1.

**Supplementary References**

Barratt, E. S. (1994). "Impulsiveness and aggression," in *Violence and Mental Disorder: Developments in Risk Assessment*, eds. J. Monahan and H. J. Steadman (Chicago IL: University of Chicago Press), 61-79.

Carver, C. S., and White, T. L. (1994). Behavioral inhibition, behavioral activation, and affective responses to impending reward and punishment: The BIS/BAS scales. *J. Pers. Soc. Psychol.* 67, 319–333. doi: 10.1037/0022-3514.67.2.319

Desikan R. S., Segonne F., Fischl B., Quinn B. T., Dickerson B. C., Blacker D., et al. (2006). An automated labeling system for subdividing the human cerebral cortex on MRI scans into gyral based regions of interest. *Neuroimage* 31, 968–980. doi: 10.1016/j.neuroimage.2006.01.021

Hyler, S. E. (1994). Personality Diagnostic Questionnaire-4+. New York: New York State Psychiatric Institute.

Lee, B. O., Lee, C. H., Lee, P. G., Choi, M. J., and Namkoong, K. (2000). Development of Korean Version of Alcohol Use Disorders Identification Test (AUDIT-K): Its Reliability and Validity. *J. Korean. Acad. Addict. Psychiatry* 4, 83-92.

Mori, S., Crain, B. J., Chacko, V. P., and Van Zijl, P. C. (1999). Three-dimensional tracking of axonal projections in the brain by magnetic resonance imaging. *Ann. Neurol*. 45, 265-269. doi: 10.1002/1531-8249(199902)45:2<265::aid-ana21>3.0.co;2-3

Patton, J. H., Stanford, M. S., and Barratt, E. S. (1995). Factor structure of the Barratt Impulsiveness Scale. *J. Clin. Psychol*. 51, 768-774. doi: 10.1002/1097-4679(199511)51:6<768::aid-jclp2270510607>3.0.co;2-1

Rae, C. L., Correia, M. M., Altena, E., Hughes, L. E., Barker, R. A., and Rowe, J. B. (2012). White matter pathology in Parkinson's disease: the effect of imaging protocol differences and relevance to executive function. Neuroimage 62, 1675-1684. doi: 10.1016/j.neuroimage.2012.06.012

Saunders, J. B., Aasland, O. G., Babor, T. F., De la Fuente, J. R., and Grant, M. (1993). Development of the Alcohol Use Disorders Identification Test (AUDIT): WHO Collaborative Project on Early Detection of Persons with Harmful Alcohol Consumption--II. *Addiction* 88, 791-804. doi: 10.1111/j.1360-0443.1993.tb02093.x

Sorg, S. F., Taylor, M. J., Alhassoon, O. M., Gongvatana, A., Theilmann, R. J., Frank, L. R., et al. (2012). Frontal white matter integrity predictors of adult alcohol treatment outcome. Biol. Psychiatry 71, 262-268. doi: 10.1016/j.biopsych.2011.09.022

Sotiropoulos, S. N., Jbabdi, S., Xu, J., Andersson, J. L., Moeller, S., Auerbach, E. J., et al. (2013). Advances in diffusion MRI acquisition and processing in the Human Connectome Project. Neuroimage 80, 125-143. doi: 10.1016/j.neuroimage.2013.05.057

Spielberger, C. D. (1988). Manual for the State-Trait Anger Expression Inventory (STAXI). *Odessa, FL: Psychological Assessment Resources.*

Stricker, N. H., Schweinsburg, B. C., Delano-Wood, L., Wierenga, C. E., Bangen, K. J., Haaland, K. Y., et al. (2009). Decreased white matter integrity in late-myelinating fiber pathways in Alzheimer's disease supports retrogenesis. Neuroimage 45, 10-16. doi: 10.1016/j.neuroimage.2008.11.027

Wiech, K., Jbabdi, S., Lin, C. S., Andersson, J., and Tracey, I. (2014). Differential structural and resting state connectivity between insular subdivisions and other pain-related brain regions. Pain 155, 2047-2055. doi: 10.1016/j.pain.2014.07.009

Yamada, H., Abe, O., Shizukuishi, T., Kikuta, J., Shinozaki, T., Dezawa, K., et al. (2014). Efficacy of distortion correction on diffusion imaging: comparison of FSL eddy and eddy_correct using 30 and 60 directions diffusion encoding. PloS One 9, e112411. doi: 10.1371/journal.pone.0112411

Zorlu, N., Çapraz, N., Oztekin, E., Bagci, B., Di Biase, M. A., Zalesky, A., et al. (2019). Rich club and reward network connectivity as endophenotypes for alcohol dependence: a diffusion tensor imaging study. Addict. Biol. 24, 265-274. doi: 10.1111/adb.12599

**Supplementary Figures**

| 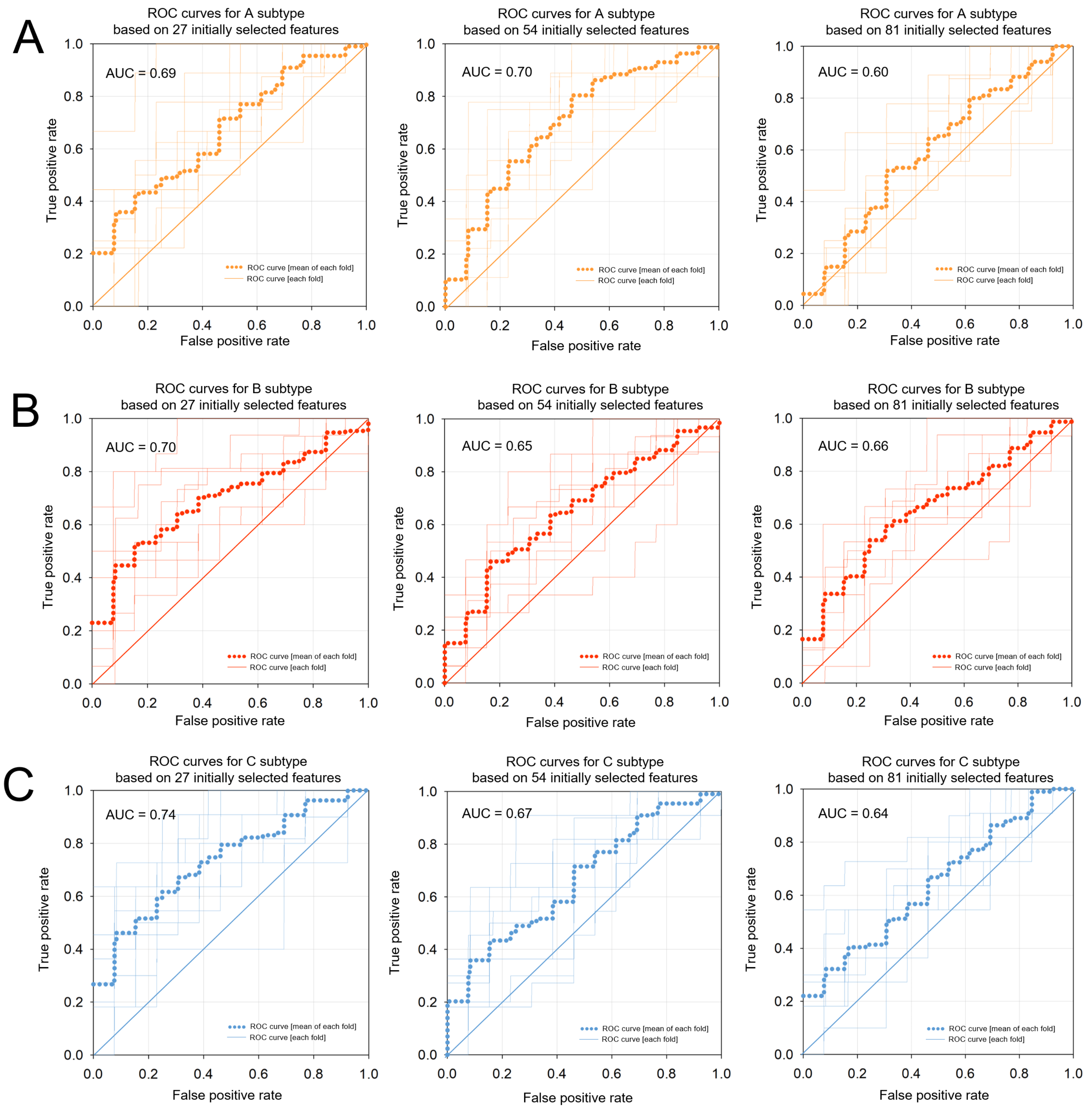 |
| --- |
| **Figure S1.** ROC curves of the classification models for identifying each subtype according to the different feature sets initially selected. Three distinct criteria for the initial feature reduction step were applied, where the top 10% (left column), 20% (middle column), and 30% (right column), respectively, of connection features with the greatest between-group differences were selected: (A) neutral vs. A subtypes; (B) neutral vs. B subtypes; (C) neutral vs. C subtypes. Thin lines within each ROC graph indicate the ROC curve from each outer fold test set, and dotted lines were obtained by averaging the ROC curves from the outer 10-fold test sets. ROC, receiver operating characteristic; AUC, area under the curve |

| 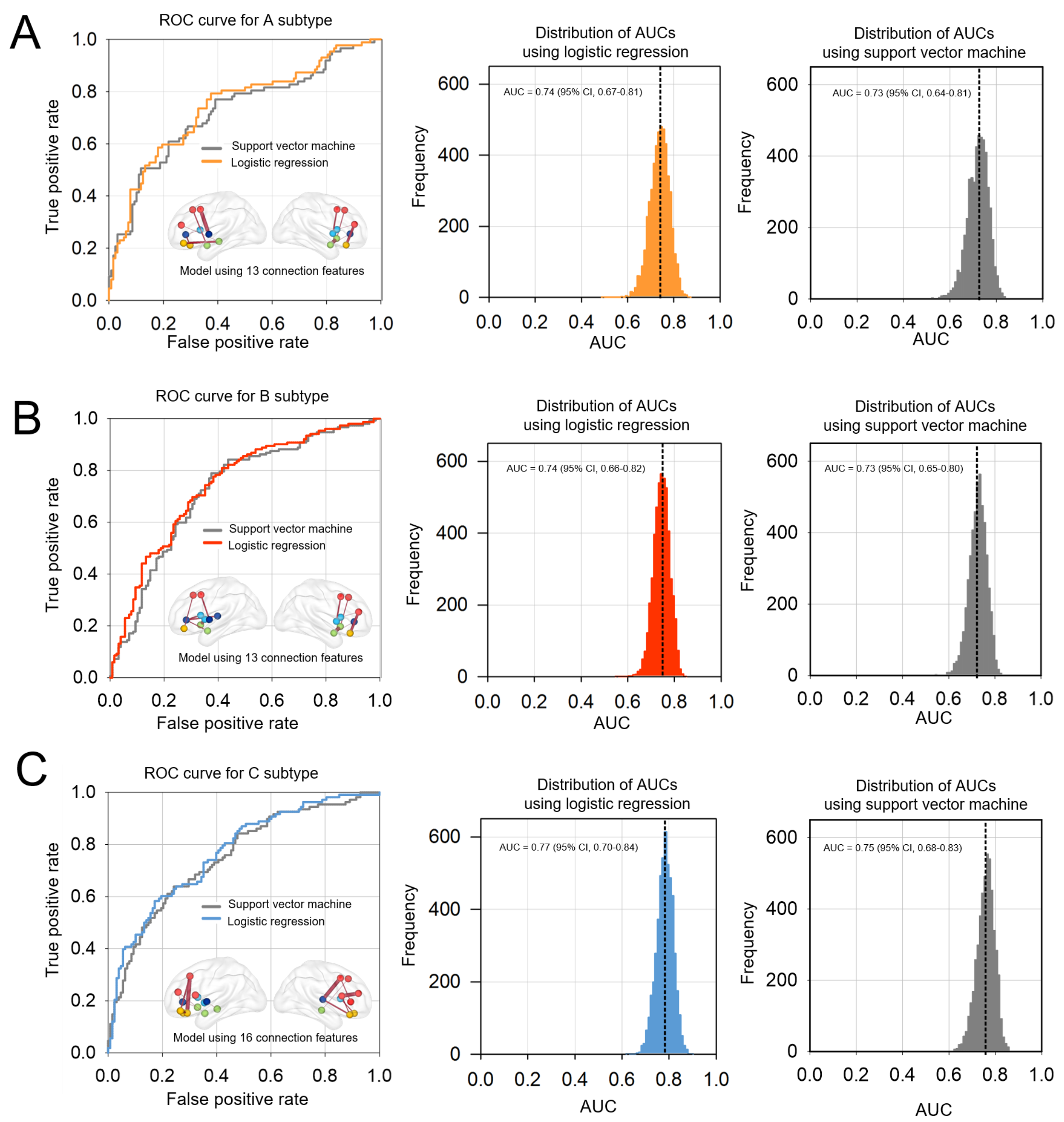 |
| --- |
| **Figure S2.** ROC curves using the regularized logistic regression and linear support vector machine methods based on the top 30% of the most frequently selected connection features for predicting the A subtype **(A)**, B subtype **(B)**, and C subtype **(C)** relative to the neutral subtype. Histograms of areas under the ROC curves (AUC) using a bootstrapping procedure with a resampling of 5,000 times are also presented, where the dashed black lines indicate the AUC for classifying each subtype apart from the neutral type.  ROC, receiver operating characteristic. |

**3. Supplementary Tables**

| **Table S1.** Regions of interest (ROIs) within the addiction circuitry that were investigated in the current study | |
| --- | --- |
| ROIs | Individual nodes clustered into each ROI, bilaterally^a^ |
| Dorsolateral prefrontal cortical (DLPFC) region | Superior frontal cortex |
|  | Rostral middle frontal cortex |
|  | Caudal middle frontal cortex |
|  | Inferior frontal cortex, pars opercularis |
|  | Inferior frontal cortex, pars triangularis |
| Orbitofrontal cortical (OFC) region | Lateral orbitofrontal cortex |
|  | Medial orbitofrontal cortex |
|  | Inferior frontal cortex, pars orbitalis |
| Limbic region | Hippocampus |
|  | Amygdala |
|  | Nucleus accumbens |
| Dorsal striatal region | Caudate |
|  | Putamen |
|  | Pallidum |
| Interoceptive salience processing region | Thalamus |
|  | Insula |
|  | Anterior cingulate cortex |
| ^a^The regions were parcellated using the FreeSurfer tool (http://surfer.nmr.mgh.harvard.edu) based on the high-resolution T1-weighted image of each individual. | |

| **Table S2.** Normalized connections interconnecting the 5 ROI groups for each subtype | | | | |
| --- | --- | --- | --- | --- |
|  | Neutral subtype | A subtype | B subtype | C subtype |
| No. of individuals | 128 | 87 | 152 | 108 |
| Right hemisphere |  |  |  |  |
| DLPFC region | 0.144 (0.014) | 0.147 (0.013) | 0.147 (0.014) | 0.150 (0.015)^a^ |
| OFC region | 0.088 (0.010) | 0.091 (0.012)^a^ | 0.090 (0.011) | 0.089 (0.011) |
| Limbic region | 0.064 (0.011) | 0.068 (0.010)^a^ | 0.064 (0.012) | 0.064 (0.012) |
| Dorsal striatal region | 0.104 (0.013) | 0.104 (0.012) | 0.102 (0.012) | 0.105 (0.012) |
| Interoceptive processing region | 0.092 (0.010) | 0.089 (0.010)^a^ | 0.092 (0.011) | 0.092 (0.010) |
| Left hemisphere |  |  |  |  |
| DLPFC region | 0.155 (0.014) | 0.153 (0.013) | 0.156 (0.014) | 0.156 (0.014) |
| OFC region | 0.093 (0.012) | 0.093 (0.011) | 0.094 (0.011) | 0.092 (0.011) |
| Limbic region | 0.062 (0.012) | 0.062 (0.011) | 0.059 (0.011)^b^ | 0.059 (0.012)^b^ |
| Dorsal striatal region | 0.099 (0.011) | 0.099 (0.010) | 0.099 (0.011) | 0.098 (0.012) |
| Interoceptive processing region | 0.098 (0.012) | 0.095 (0.010) | 0.097 (0.011) | 0.096 (0.012) |
| ^a^The statistical significance at permutation-adjusted *P* < 0.05 in normalized connections of each subtype as compared with those of the neutral subtype.  ^b^The marginal statistical significance of between-group differences at permutation-adjusted *P* < 0.09.  No, number; DLPFC, dorsolateral prefrontal cortex; OFC, orbitofrontal cortex. | | | | |

| **Table S3.** The list of the 54 remaining connection feature candidates after the initial reduction of features | | | | | | | |
| --- | --- | --- | --- | --- | --- | --- | --- |
| Neutral vs. A subtypes | |  | Neutral vs. B subtypes | |  | Neutral vs. C subtypes | |
| Connection feature candidates | |  | Connection feature candidates | |  | Connection feature candidates | |
| R | cMFC-pars triangularis |  | R | rMFC-LOFC |  | R | MOFC-caudate |
| R | Pars orbitalis-putamen |  | R | Amygdala-nucleus accumbens |  | R | rMFC-pars opercularis |
| R | Pars orbitalis-pallidum |  | L | Nucleus accumbens-pallidum |  | R | rMFC-LOFC |
| L | ACC-nucleus accumbens |  | R | rMFC-caudate |  | L | Pars triangularis-putamen |
| L | rMFC-cMFC |  | L | SFC-pars orbitalis |  | L | SFC-pars orbitalis |
| L | LOFC-ACC |  | L | Amygdala-nucleus accumbens |  | R | cMFC-thalamus |
| L | cMFC-insula |  | L | Insula-hippocampus |  | R | Insula-putamen |
| R | LOFC-putamen |  | R | Pars orbitalis-pallidum |  | R | Hippocampus-amygdala |
| L | Insula-hippocampus |  | L | cMFC-insula |  | L | SFC-thalamus |
| R | putamen-pallidum |  | L | rMFC-MOFC |  | R | Pars triangularis-insula |
| L | Caudate-thalamus |  | R | rMFC-ACC |  | L | Pars orbitalis-LOFC |
| R | SFC-ACC |  | R | SFC-ACC |  | L | Pars opercularis-caudate |
| L | Nucleus accumbens-thalamus |  | R | Caudate-pallidum |  | L | ACC-nucleus accumbens |
| L | rMFC-MOFC |  | R | cMFC-putamen |  | R | rMFC-ACC |
| L | Pars triangularis-LOFC |  | R | Hippocampus-putamen |  | R | MOFC-thalamus |
| L | Putamen-pallidum |  | L | rMFC-pars opercularis |  | R | SFC-pars triangularis |
| R | SFC-thalamus |  | L | Caudate-thalamus |  | L | cMFC-Insula |
| L | Pars opercularis-caudate |  | L | Pars orbitalis-pallidum |  | L | SFC-LOFC |
| L | rMFC-hippocampus |  | R | rMFC-putamen |  | R | LOFC-thalamus |
| L | Pars orbitalis-hippocampus |  | R | Pars opercularis-LOFC |  | L | Insula-hippocampus |
| R | Hippocampus-nucleus accumbens |  | L | Amygdala-putamen |  | L | LOFC-pallidum |
| L | SFC-LOFC |  | R | Pars opercularis-Pars orbitalis |  | R | SFC-ACC |
| R | MOFC-pallidum |  | L | Nucleus accumbens-putamen |  | L | rMFC-thalamus |
| R | Nucleus accumbens-thalamus |  | L | Pars orbitalis-hippocampus |  | L | Pars opercularis-pallidum |
| R | Pars orbitalis-thalamus |  | L | MOFC-amygdala |  | L | Hippocampus-pallidum |
| L | Pars orbitalis-caudate |  | R | Pars orbitalis-putamen |  | L | Amygdala-nucleus accumbens |
| L | Hippocampus-caudate |  | R | Putamen-thalamus |  | L | LOFC-putamen |
| R | cMFC-thalamus |  | R | Nucleus accumbens-caudate |  | R | LOFC-pallidum |
| L | SFC-pars opercularis |  | R | SFC-putamen |  | R | SFC-pars orbitalis |
| L | SFC-insula |  | L | rMFC-cMFC |  | L | Pars opercularis-thalamus |
| R | rMFC-caudate |  | L | MOFC-hippocampus |  | L | LOFC-ACC |
| L | rMFC-LOFC |  | L | SFC-putamen |  | L | MOFC-hippocampus |
| R | SFC-nucleus accumbens |  | L | Hippocampus-putamen |  | R | SFC-insula |
| L | Insula-pallidum |  | R | Pars opercularis-caudate |  | L | Pars opercularis-insula |
| R | rMFC-LOFC |  | R | Insula-nucleus accumbens |  | L | rMFC-hippocampus |
| L | Pars orbitalis-thalamus |  | R | SFC-pars orbitalis |  | R | rMFC-amygdala |
| R | Insula-thalamus |  | R | LOFC-putamen |  | L | SFC-mOFC |
| R | Pars opercularis-thalamus |  | R | rMFC-insula |  | L | rMFC-amygdala |
| L | Pars triangularis-pallidum |  | L | Pars triangularis-caudate |  | R | SFC-hippocampus |
| L | MOFC-pallidum |  | R | rMFC-nucleus accumbens |  | L | rMFC-pars orbitalis |
| L | MOFC-insula |  | R | ACC-amygdala |  | L | Hippocampus-amygdala |
| L | Amygdala-thalamus |  | L | Pars triangularis-putamen |  | R | SFC-amygdala |
| R | cMFC-pallidum |  | R | SFC-nucleus accumbens |  | R | Pars opercularis-LOFC |
| L | ACC-caudate |  | R | ACC-thalamus |  | L | Pars opercularis-putamen |
| R | LOFC-hippocampus |  | L | ACC-amygdala |  | R | Caudate-pallidum |
| L | SFC-amygdala |  | R | SFC-thalamus |  | L | Hippocampus-caudate |
| L | Pars triangularis-hippocampus |  | L | ACC-caudate |  | L | rMFC_ACC |
| R | SFC-pars triangularis |  | L | ACC-thalamus |  | R | cMFC-pars triangularis |
| R | Pars orbitalis-insula |  | R | ACC-hippocampus |  | L | rMFC-MOFC |
| L | Insula-caudate |  | R | cMFC-pallidum |  | R | Hippocampus-nucleus accumbens |
| L | cMFC-thalamus |  | R | Amygdala-thalamus |  | R | Pallidum-thalamus |
| R | SFC-pars orbitalis |  | R | LOFC-thalamus |  | R | LOFC-caudate |
| L | LOFC-thalamus |  | L | Pars triangularis-insula |  | L | LOFC-hippocampus |
| R | Amygdala-thalamus |  | R | Hippocampus-amygdala |  | L | Pars triangularis-pars orbitalis |
| MOFC, medial orbitofrontal cortex; cMFC, caudal middle frontal cortex; LOFC, lateral orbitofrontal cortex; SFC, superior frontal cortex; ACC, anterior cingulate cortex; rMFC, rostral middle frontal cortex. | | | | | | | |

| **Table S4.** Group comparisons of normalized degrees of individual nodes of interest | | | | | | |
| --- | --- | --- | --- | --- | --- | --- |
|  | Neutral vs. A subtypes | | Neutral vs. B subtypes | | Neutral vs. C subtypes | |
|  | *z* | P value | *z* | P value | *z* | P value |
| Right hemisphere |  |  |  |  |  |  |
| Superior frontal cortex | -0.06 | 0.95 | -0.99 | 0.32 | 0.54 | 0.59 |
| Rostral middle frontal cortex | 0.58 | 0.56 | 1.08 | 0.28 | 1.64 | 0.10 |
| Caudal middle frontal cortex | 1.81 | 0.07 | 0.20 | 0.84 | 1.28 | 0.20 |
| Inferior frontal cortex, pars opercularis | 0.40 | 0.69 | 1.66 | 0.10 | 0.21 | 0.84 |
| Inferior frontal cortex, pars triangularis | 1.74 | 0.08 | 1.78 | 0.08 | 1.37 | 0.17 |
| Lateral orbitofrontal cortex | 2.76 | 0.006 | 2.30 | 0.02 | 1.55 | 0.12 |
| Medial orbitofrontal cortex | -0.01 | 0.99 | -0.11 | 0.92 | -0.41 | 0.68 |
| Inferior frontal cortex, pars orbitalis | 0.85 | 0.39 | -0.74 | 0.46 | -0.12 | 0.90 |
| Hippocampus | 1.19 | 0.23 | 1.20 | 0.23 | -0.45 | 0.65 |
| Amygdala | 0.33 | 0.74 | -1.21 | 0.23 | 0.02 | 0.98 |
| Nucleus accumbens | 2.75 | 0.006 | -0.02 | 0.98 | 0.33 | 0.74 |
| Caudate | -0.79 | 0.43 | -0.33 | 0.74 | -0.29 | 0.77 |
| Putamen | -0.65 | 0.52 | -2.19 | 0.03 | 0.11 | 0.91 |
| Pallidum | 0.65 | 0.52 | 0.08 | 0.93 | 1.09 | 0.28 |
| Thalamus | -0.67 | 0.51 | 0.39 | 0.70 | -0.88 | 0.38 |
| Insula | -1.89 | 0.06 | -0.95 | 0.34 | 0.63 | 0.53 |
| Anterior cingulate cortex | -1.53 | 0.13 | 0.57 | 0.57 | 0.62 | 0.53 |
| Left hemisphere |  |  |  |  |  |  |
| Superior frontal cortex | -0.24 | 0.81 | 0.67 | 0.51 | 1.88 | 0.06 |
| Rostral middle frontal cortex | -0.36 | 0.72 | -0.80 | 0.42 | -1.82 | 0.07 |
| Caudal middle frontal cortex | 1.12 | 0.26 | 0.27 | 0.79 | 1.47 | 0.14 |
| Inferior frontal cortex, pars opercularis | -1.89 | 0.06 | -0.79 | 0.43 | -1.66 | 0.10 |
| Inferior frontal cortex, pars triangularis | -1.01 | 0.31 | 1.28 | 0.20 | 0.70 | 0.48 |
| Lateral orbitofrontal cortex | -0.22 | 0.82 | 0.22 | 0.82 | -0.91 | 0.36 |
| Medial orbitofrontal cortex | 0.32 | 0.75 | 0.22 | 0.83 | -0.75 | 0.45 |
| Inferior frontal cortex, pars orbitalis | -0.67 | 0.50 | 0.35 | 0.72 | 0.09 | 0.93 |
| Hippocampus | 0.19 | 0.85 | 0.47 | 0.64 | -0.73 | 0.46 |
| Amygdala | -0.31 | 0.75 | -2.67 | 0.008 | -1.75 | 0.08 |
| Nucleus accumbens | -0.16 | 0.87 | -1.28 | 0.20 | -1.04 | 0.30 |
| Caudate | -0.38 | 0.70 | -0.63 | 0.53 | -1.44 | 0.15 |
| Putamen | 0.15 | 0.88 | 0.15 | 0.88 | 0.74 | 0.46 |
| Pallidum | -0.28 | 0.78 | 0.22 | 0.83 | -0.82 | 0.42 |
| Thalamus | -1.67 | 0.10 | -0.20 | 0.84 | -0.71 | 0.48 |
| Insula | -1.51 | 0.13 | -0.87 | 0.38 | -1.04 | 0.30 |
| Anterior cingulate cortex | -0.07 | 0.95 | -0.01 | 0.99 | 0.39 | 0.70 |
| The general linear model was used to compare the normalized degree of each node between the groups after adjusting for sex. | | | | | | |

| **Table S5.** The list and frequency of the selected features that appeared in over the 40% of the repeated cross-validation trials | | | | | | | | | | |
| --- | --- | --- | --- | --- | --- | --- | --- | --- | --- | --- |
| Neutral vs. A subtypes | | |  | Neutral vs. B subtypes | | |  | Neutral vs. C subtypes | | |
| Selected features | | Frequency (%) |  | Selected features | | Frequency (%) |  | Selected features | | Frequency (%) |
| R | MOFC-pallidum | 90 |  | R | Amygdala-nucleus accumbens | 100 |  | R | rMFC-pars opercularis | 100 |
| L | cMFC-insula | 90 |  | L | Nucleus accumbens-pallidum | 90 |  | R | cMFC-thalamus | 100 |
| R | LOFC-putamen | 80 |  | R | rMFC-LOFC | 80 |  | L | SFC-LOFC | 100 |
| R | cMFC-pars triangularis | 80 |  | R | cMFC-putamen | 60 |  | L | Pars orbitalis-LOFC | 70 |
| R | Insula-thalamus | 70 |  | L | ACC-thalamus | 60 |  | R | LOFC-thalamus | 60 |
| L | Pars orbitalis-hippocampus | 70 |  | L | cMFC-insula | 60 |  | L | SFC-pars orbitalis | 60 |
| R | SFC-nucleus accumbens | 60 |  | L | ACC-caudate | 60 |  | R | MOFC-caudate | 50 |
| R | cMFC-thalamus | 60 |  | R | rMFC-ACC | 50 |  | R | cMFC-pars triangularis | 50 |
| L | LOFC-ACC | 60 |  | R | SFC-nucleus accumbens | 50 |  | R | Pars opercularis-LOFC | 50 |
| L | SFC-amygdala | 60 |  | L | SFC-pars orbitalis | 50 |  | L | Pars opercularis-caudate | 50 |
| R | hippocampus-nucleus accumbens | 50 |  | L | ACC-amygdala | 50 |  | R | SFC-hippocampus | 40 |
| L | rMFC-hippocampus | 50 |  | R | rMFC-caudate | 40 |  | L | Insula-hippocampus | 40 |
| L | ACC-caudate | 50 |  | R | ACC-amygdala | 40 |  | L | Pars opercularis-pallidum | 40 |
| R | Pars orbitalis-pallidum | 40 |  |  |  |  |  | L | rMFC-MOFC | 40 |
| R | Pars opercularis-thalamus | 40 |  |  |  |  |  | L | rMFC-amygdala | 40 |
| R | LOFC-hippocampus | 40 |  |  |  |  |  | L | ACC-nucleus accumbens | 40 |
| L | SFC-LOFC | 40 |  |  |  |  |  |  |  |  |
| L | MOFC-pallidum | 40 |  |  |  |  |  |  |  |  |
| L | MOFC-insula | 40 |  |  |  |  |  |  |  |  |
| L | Insula-hippocampus | 40 |  |  |  |  |  |  |  |  |
| Abbreviations: MOFC, medial orbitofrontal cortex; cMFC, caudal middle frontal cortex; LOFC, lateral orbitofrontal cortex; SFC, superior frontal cortex; ACC, anterior cingulate cortex; rMFC, rostral middle frontal cortex | | | | | | | | | | |
